# Supplementary material for: Network pharmacology approach identifies novel anticancer botanicals: Experimental exploration of Falcaria vulgaris (Sickleweed) as a therapeutic candidate
Source: PLoS One. 2026 Feb 27;21(2):e0334417. doi: 10.1371/journal.pone.0334417 (PMC12948079; doi:10.1371/journal.pone.0334417)
Supplement: S1 Fig — Venn diagram analysis showed the number of known anticancer metabolites in each top plant which were listed in the table of metabolites. (DOCX) [file pone.0334417.s001.docx]

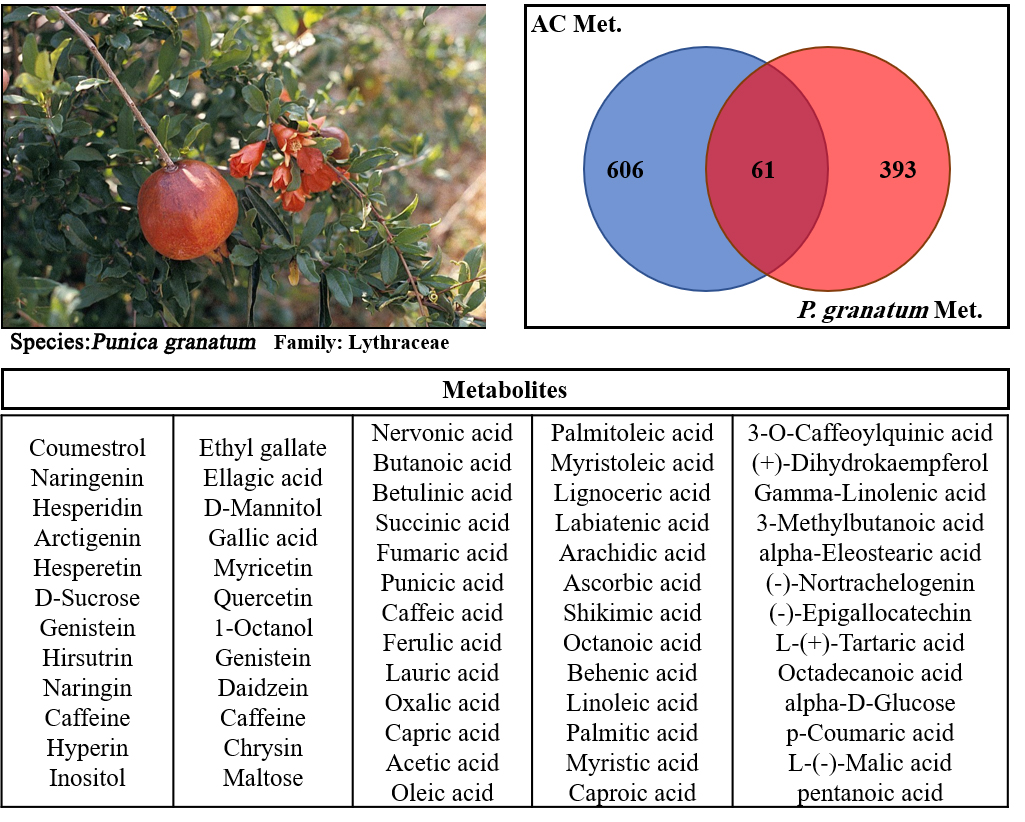


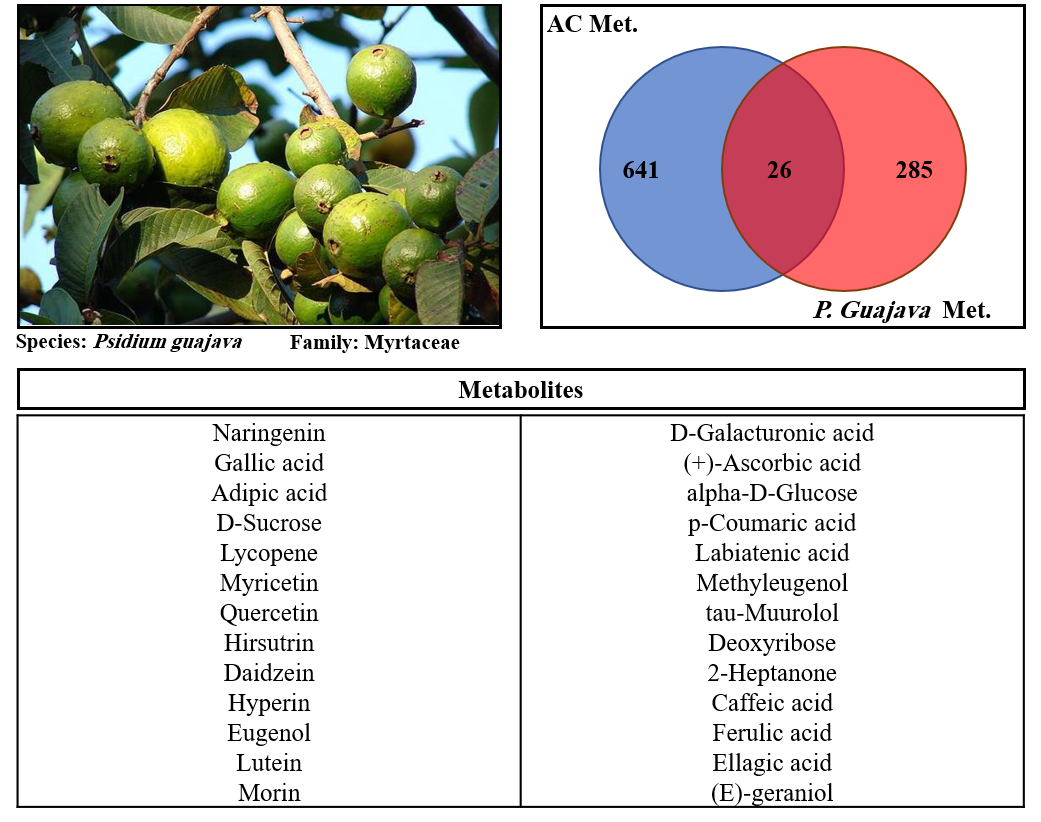


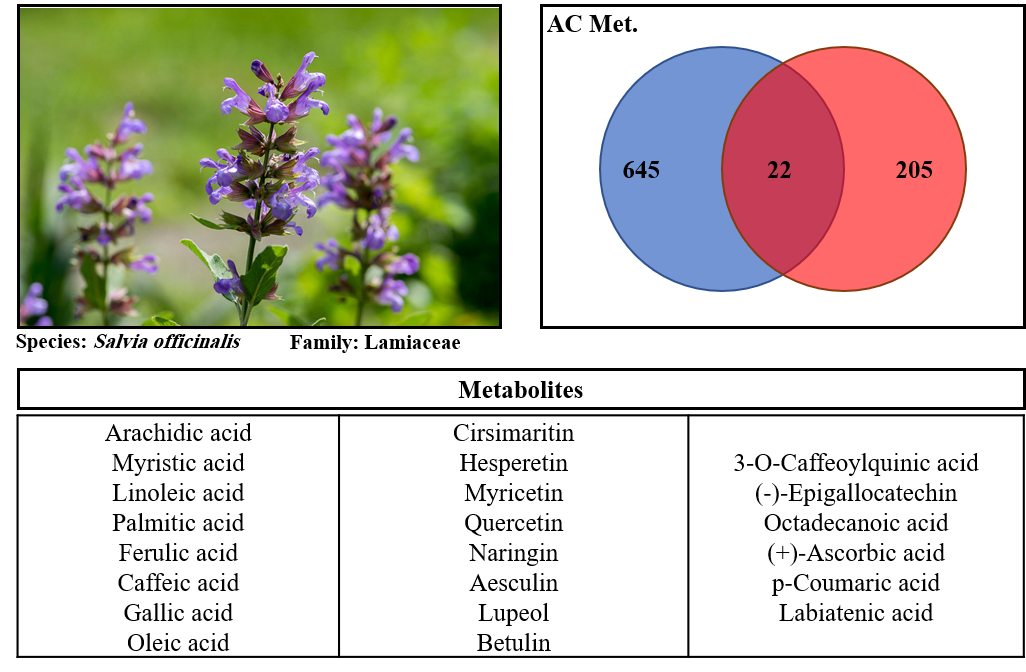


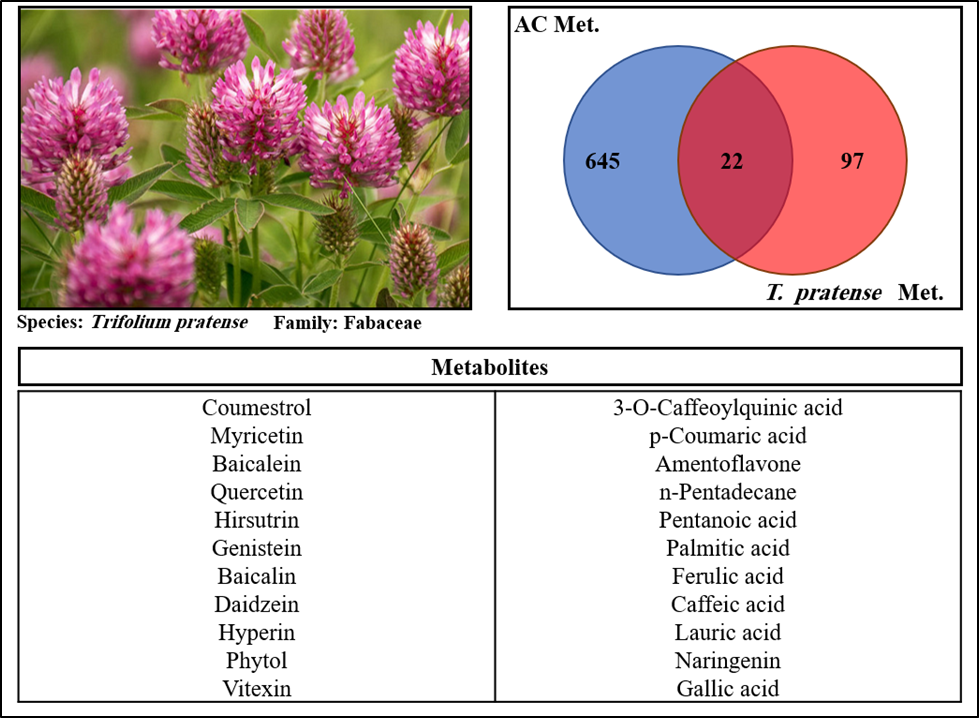


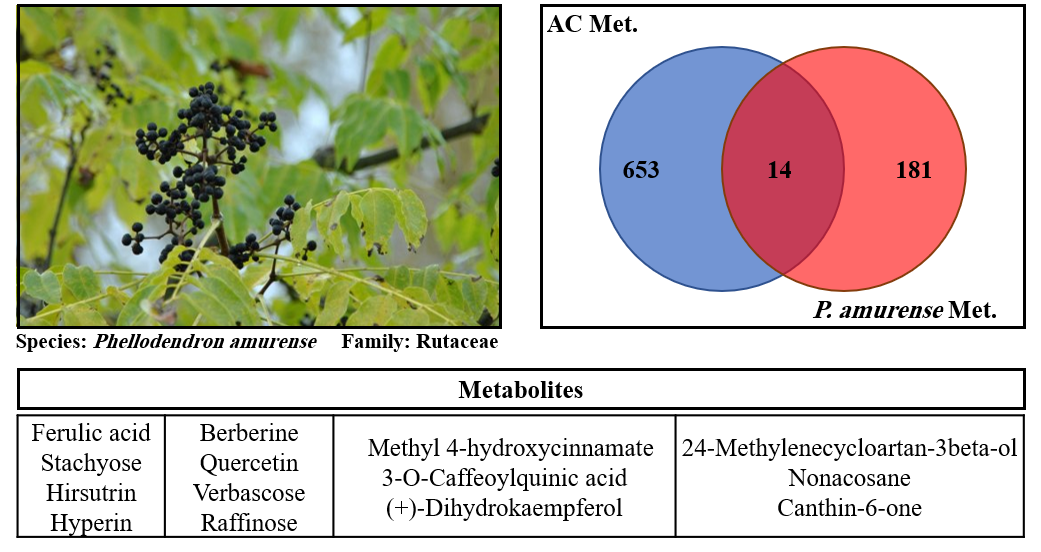


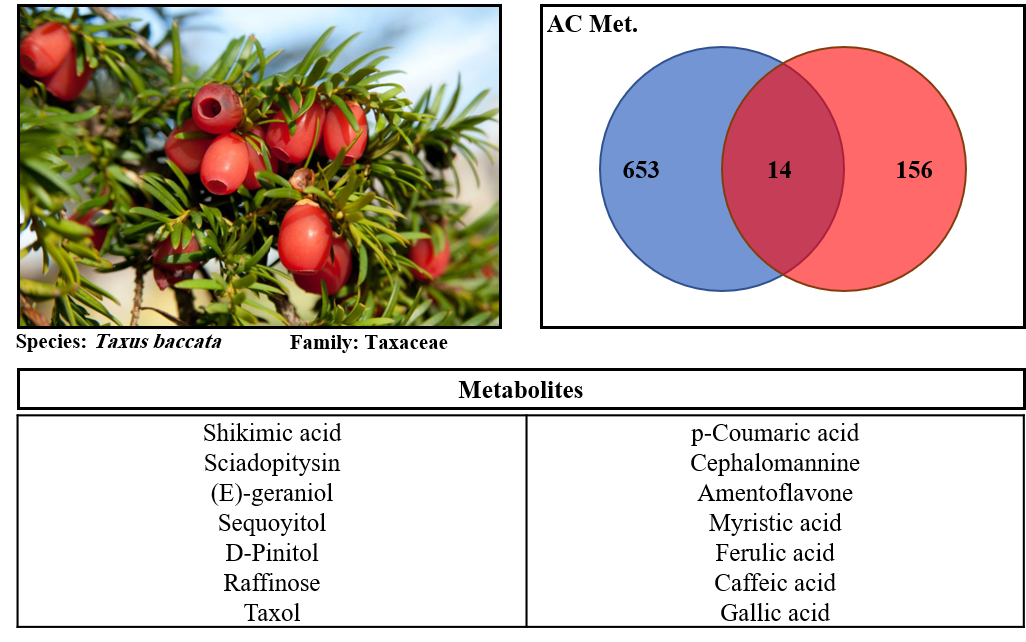


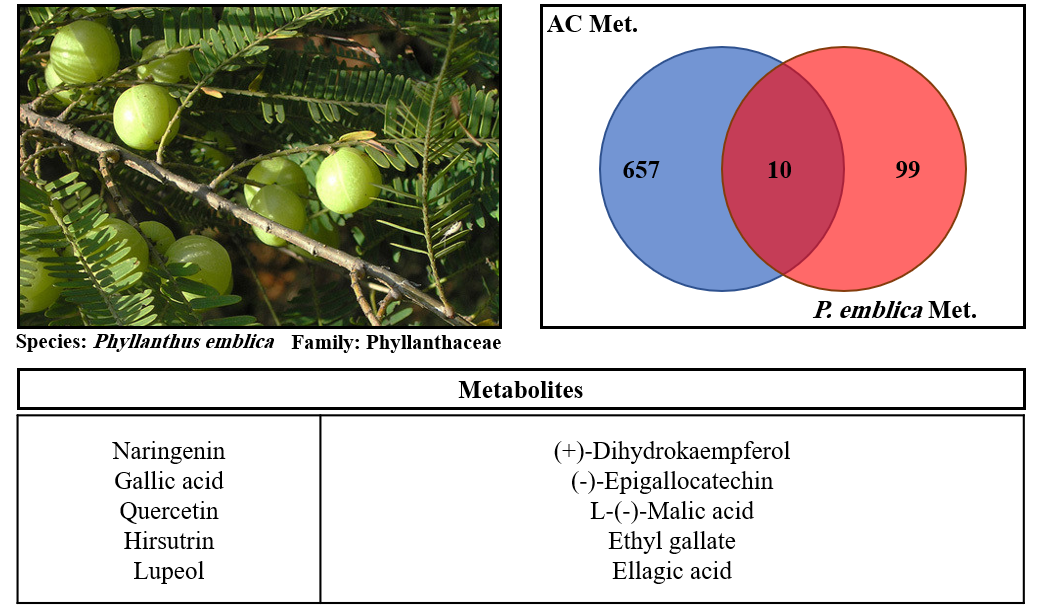


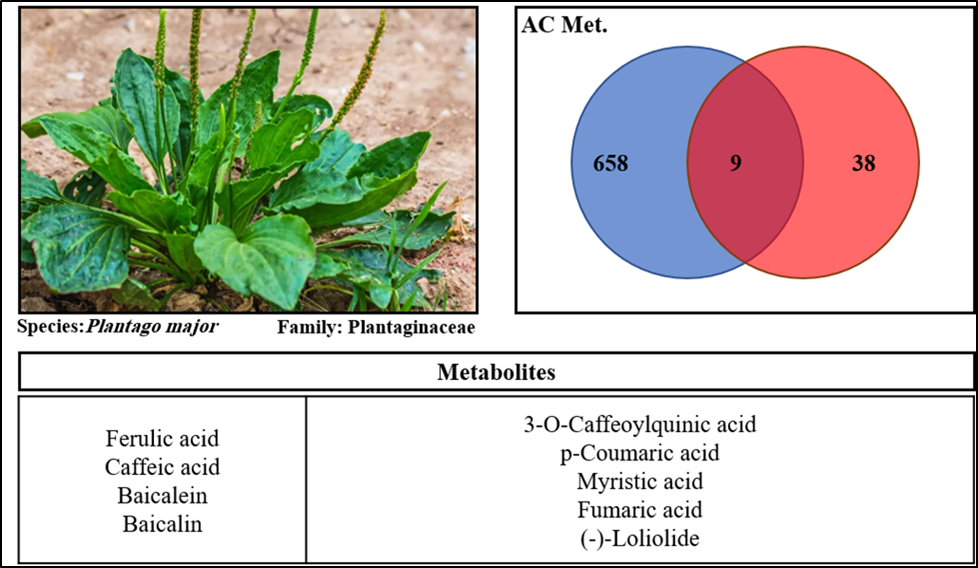


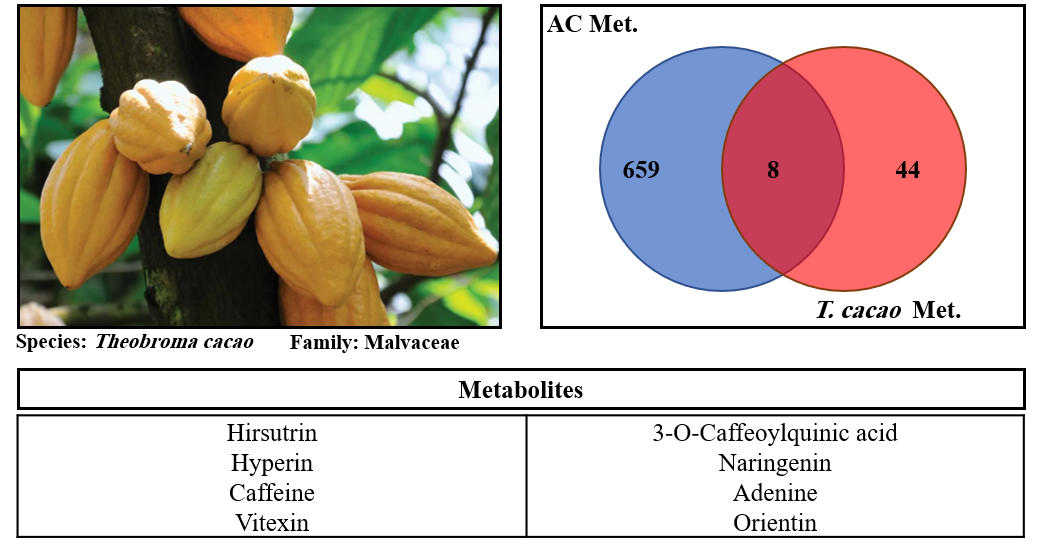


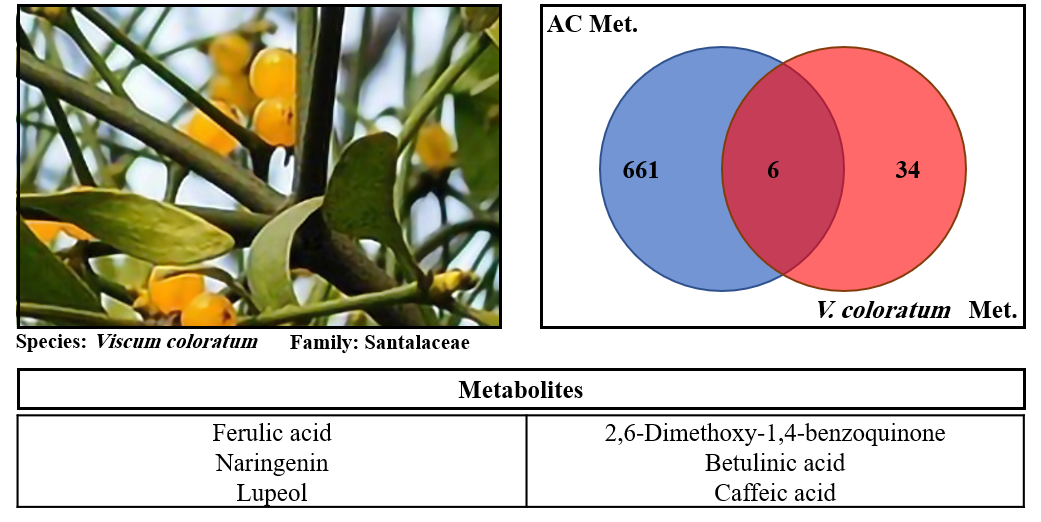


**S1 Fig.** The detailed information of top ten plants with the highest replication that their anti-cancer properties were previously established. Venn diagram analysis showed the number of known anti-cancer metabolites in each top plant which were listed in the table of metabolites.
