# Supplementary material for: Network pharmacology approach identifies novel anticancer botanicals: Experimental exploration of Falcaria vulgaris (Sickleweed) as a therapeutic candidate
Source: PLoS One. 2026 Feb 27;21(2):e0334417. doi: 10.1371/journal.pone.0334417 (PMC12948079; doi:10.1371/journal.pone.0334417)
Supplement: S2 Fig — Venn diagram analysis showed the number of known anticancer metabolites in those plants. (DOCX) [file pone.0334417.s002.docx]

| Plant species photo | Venn diagram analysis | Plant species information |
| --- | --- | --- |
| 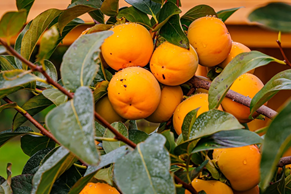 | 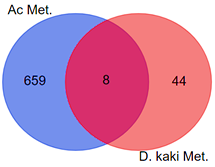 | \| Species: \| Diospyros kaki \| \| --- \| --- \| \| Family: \| Ebenaceae \| \| Replicate: \| 8 \| |
| 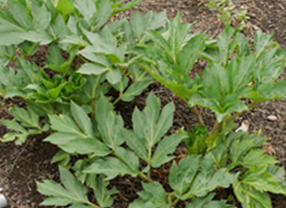 | 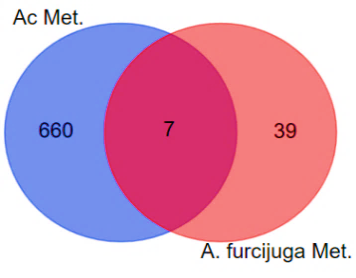 | \| Species: \| Angelica furcijuga \| \| --- \| --- \| \| Family: \| Apiaceae \| \| Replicate: \| 6 \| |
| 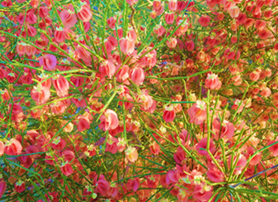 | 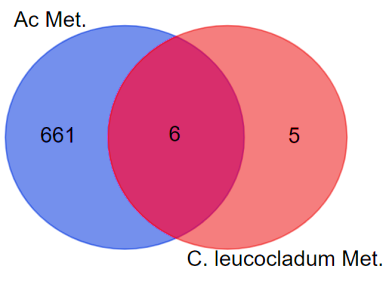 | \| Species: \| Calligonum leucocladum \| \| --- \| --- \| \| Family: \| Polygonaceae \| \| Replicate: \| 6 \| |
| 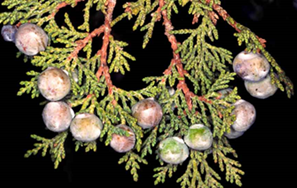 | 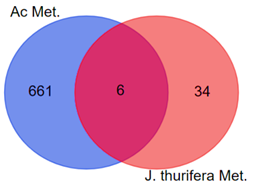 | \| Species: \| Juniperus thurifera \| \| --- \| --- \| \| Family: \| [Cupressaceae](https://en.wikipedia.org/wiki/Cupressaceae) \| \| Replicate: \| 5 \| |
| 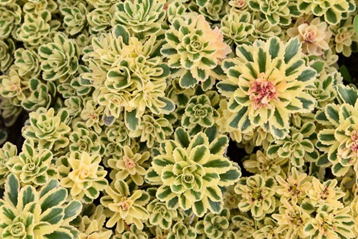 | 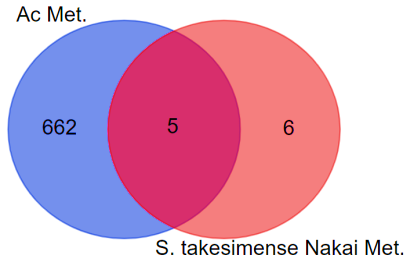 | \| Species: \| Sedum takesimense \| \| --- \| --- \| \| Family: \| Crassulaceae \| \| Replicate: \| 6 \| |
| 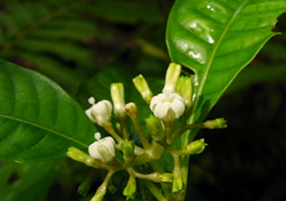 | 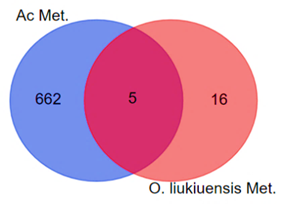 | \| Species: \| Ophiorrhiza liukiuensis \| \| --- \| --- \| \| Family: \| Rubiaceae \| \| Replicate: \| 5 \| |
| 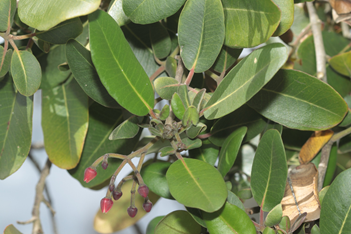 | 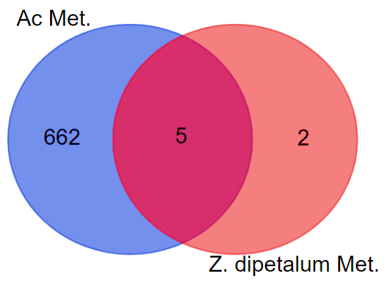 | \| Species: \| Zanthoxylum dipetalum \| \| --- \| --- \| \| Family: \| Rutaceae \| \| Replicate: \| 6 \| |
| 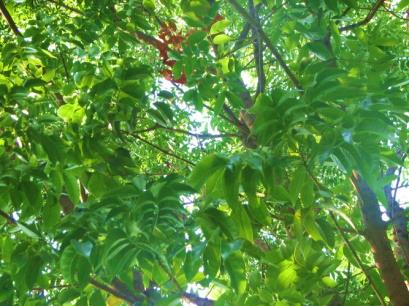 | 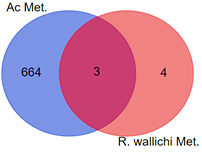 | \| Species: \| Rhus wallichii \| \| --- \| --- \| \| Family: \| Anacardiaceae \| \| Replicate: \| 5 \| |
| 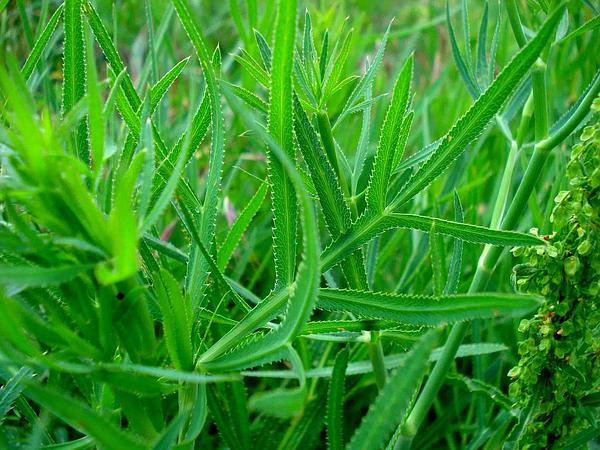 | 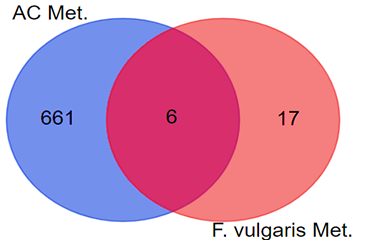 | \| Species: \| F.vulgaris \| \| --- \| --- \| \| Family: \| [Apiaceae](https://www.ipmimages.org/browse/subinfo.cfm?sub=48452) \| \| Replicate: \| 6 \| |

**S2 Fig.** The detailed information of nine potential anti-cancer plants that their anti-cancer properties were not previously established. Venn diagram analysis showed the number of known anti-cancer metabolites in those plants.
