# Supplementary figures and images for: Network pharmacology approach identifies novel anticancer botanicals: Experimental exploration of Falcaria vulgaris (Sickleweed) as a therapeutic candidate
Source: PLoS One. 2026 Feb 27;21(2):e0334417. doi: 10.1371/journal.pone.0334417 (PMC12948079; doi:10.1371/journal.pone.0334417)

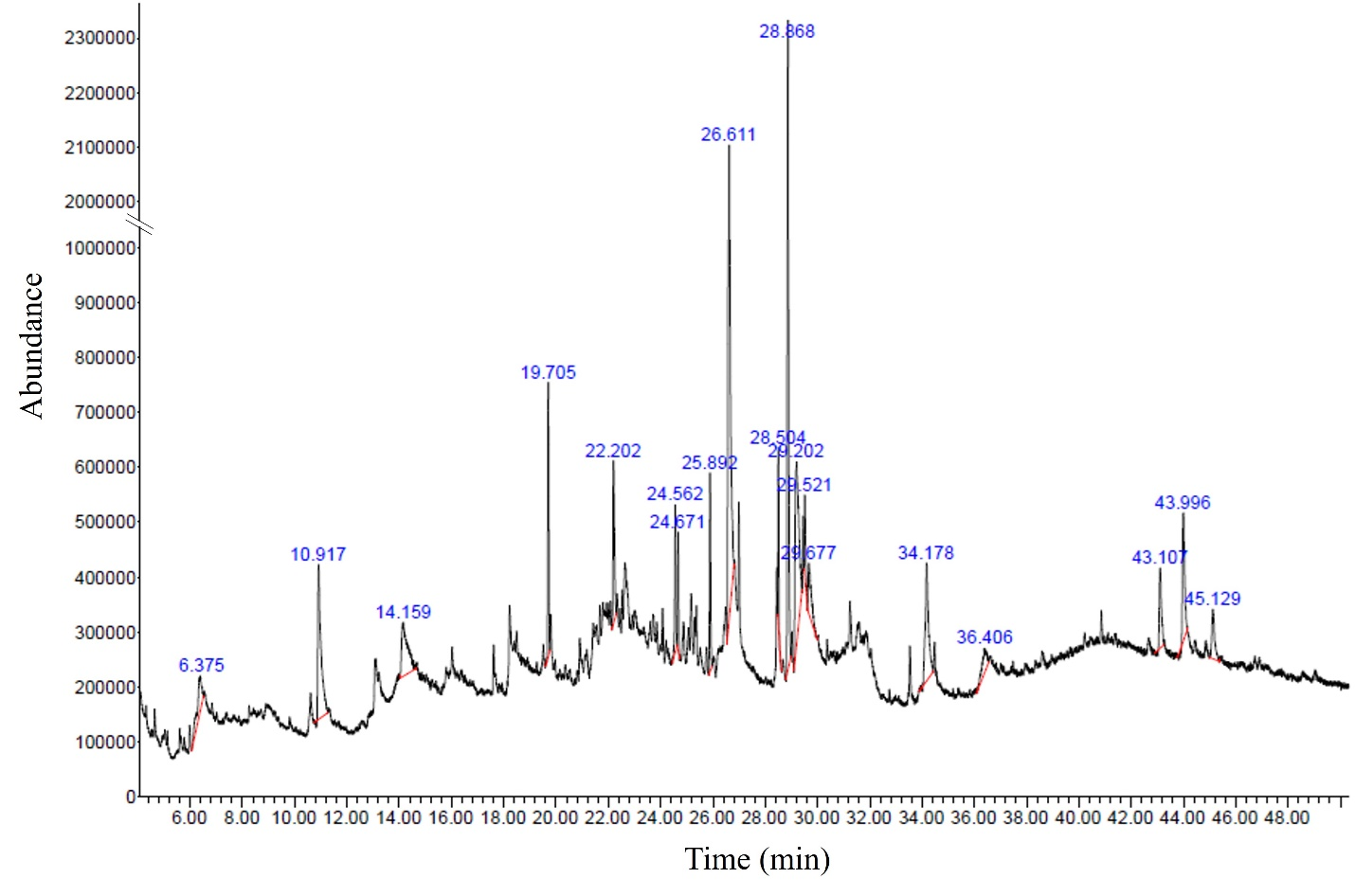

Supplement: S3 Fig — (TIF) [file pone.0334417.s003.tif]

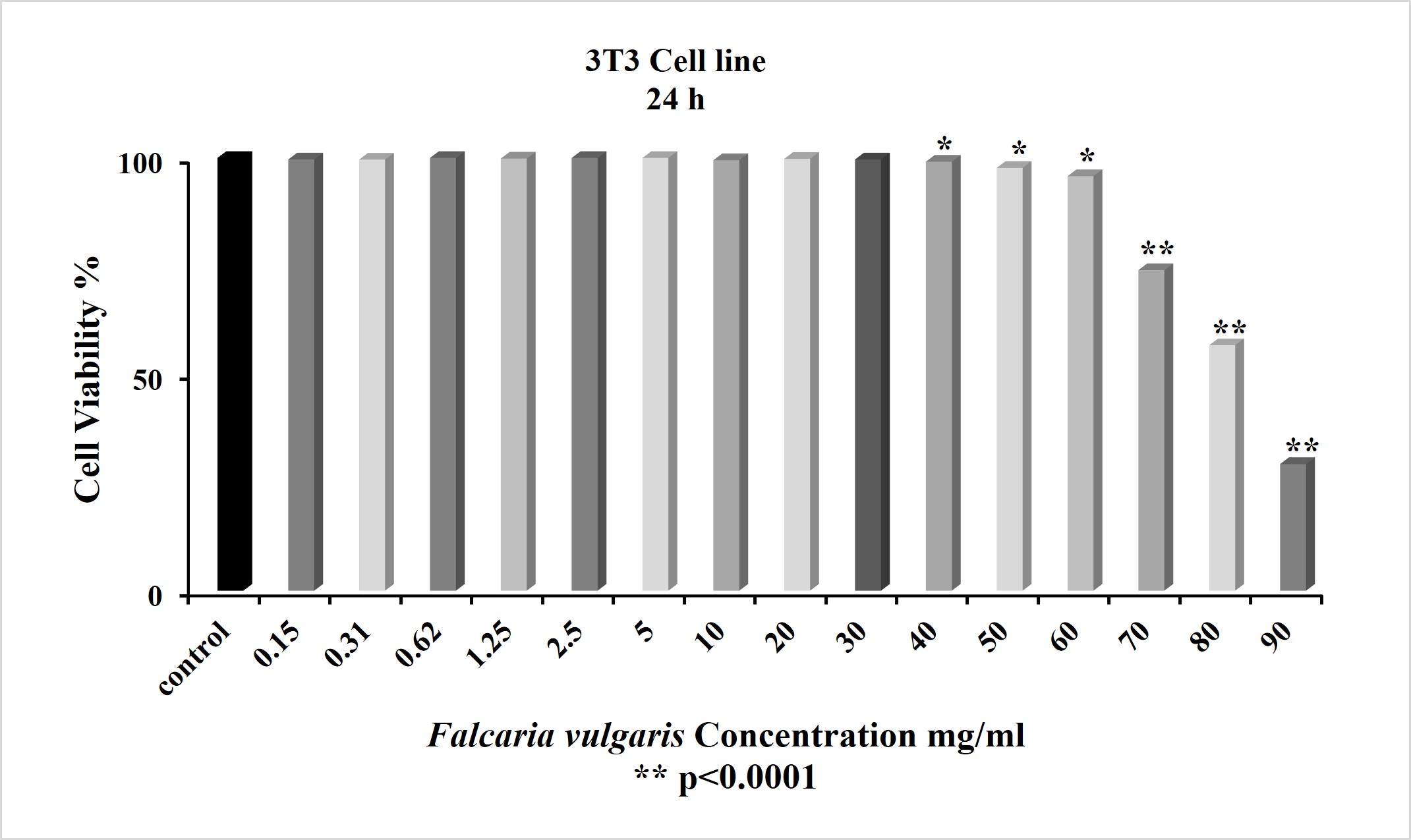

Supplement: S4 Fig — No significant cytotoxicity was observed at concentrations up to 30 mg/ml compared to the untreated control (p > 0.05). (JPG) [file pone.0334417.s004.jpg]
