# Supplementary material for: Network pharmacology approach identifies novel anticancer botanicals: Experimental exploration of Falcaria vulgaris (Sickleweed) as a therapeutic candidate
Source: PLoS One. 2026 Feb 27;21(2):e0334417. doi: 10.1371/journal.pone.0334417 (PMC12948079; doi:10.1371/journal.pone.0334417)
Supplement: S3 Table — The eight topological measures were computed using CytoHubba and Network Analyzer in Cytoscape 3.9.1. (DOCX) [file pone.0334417.s007.docx]

**S3 Table:** Complete list of network analysis parameters used for plant ranking, including mathematical formulations and calculation algorithms. The eight topological measures were computed using CytoHubba and Network Analyzer in Cytoscape 3.9.1.

| **Description** | **Equation** | **Parameter** |
| --- | --- | --- |
| Where *N(v)* is the number of the neighbors of node | *Deg(V)=* $\left\vert N(v) \right\vert$ | Degree |
| Where $\sigma_{st}$is the number of shortest paths from node *s*  to node *t* | BC(v)=$\sum_{s\neq t\neq v\in c(v)} \frac{\sigma_{st}(v)}{\sigma_{st}}$ | Betweenness |
| The top central metabolite or plant in networks | *Clo(v)=* $\sum_{w\in V} \frac{1}{dist(v, w)}$ | Closeness |
| Let $T_{s}$ be a shortest path tree rooted at node *s*. Where $p_{s}\left( v \right)=1$ if more than $\frac{\left\vert V\left( T_{s} \right) \right\vert}{4}$  Paths from node *s* to other nodes in $T_{s}$  meet at the vertex *v*; otherwise $P_{s}$(v)= 0 | *BN(v)=* $\sum_{s\in V} P_{s}$*(v)* | Bottleneck |
|  | *EC=*$\frac{\left\vert V(C\left( v \right)) \right\vert}{\left\vert V \right\vert}\times\frac{1}{max\{dist\left( v, w \right): w\in C\left( v \right)\}}$ | Eccentricity |
| $\Delta_{C\left( v \right)}$is the maximum distance between any two vertices of the component *C(v)* | *Rad(v)=* $\frac{\left\vert V(C\left( v \right)) \right\vert}{\left\vert V \right\vert}$ $\times\frac{\sum_{w\in C(v)} (\Delta_{C\left( v \right)}+1-dist(v, w)}{max\{dist\left( v, w \right):w\in c\left( v \right)\}}$ | Radiality |
| Where $\sigma_{st}(v)$  is the number of shortest paths from node *s* to node *t* which use the node *v* | *v))Str (v)=* $\sum_{s\neq t\neq v\in C(v)} \sigma_{st}$ | Stress |
| Let the $G_{k}$  be the reduced network generated at the $K_{th}$  time reduced process. If nodes *u* and *v* are connected in $G_{k}$  , set $\delta_{vt}^{k}$  To be 1; otherwise $\delta_{vt}^{k}$=0 | *EPC(v)=*$\frac{1}{\left\vert V \right\vert} \sum_{k=1}^{1000} \sum_{t\in1} \delta_{vt}^{k}$ | *EPC*  *(Edge Percolated Component)* |
