# Supplementary material for: Network pharmacology approach identifies novel anticancer botanicals: Experimental exploration of Falcaria vulgaris (Sickleweed) as a therapeutic candidate
Source: PLoS One. 2026 Feb 27;21(2):e0334417. doi: 10.1371/journal.pone.0334417 (PMC12948079; doi:10.1371/journal.pone.0334417)
Supplement: S4 Table — (DOCX) [file pone.0334417.s008.docx]

**S4 Table:** Comparative study of IC50 values for different periods of incubation for the two cell lines (MCF7, and 4T1) exposed to different concentrations of *F. vulgaris* extract (5-90 mg/ml).

| **IC50 values mg/ml** | | |
| --- | --- | --- |
| **Incubation Period, h** | **MCF7** | **4T1** |
| **24** | 5.32 | 5.99 |
| **48** | 7.02 | 9.23 |
| **72** | 7.03 | 9.19 |
