# Supplementary material for: Network pharmacology approach identifies novel anticancer botanicals: Experimental exploration of Falcaria vulgaris (Sickleweed) as a therapeutic candidate
Source: PLoS One. 2026 Feb 27;21(2):e0334417. doi: 10.1371/journal.pone.0334417 (PMC12948079; doi:10.1371/journal.pone.0334417)
Supplement: Attachment — Submitted filename: Review-PLOSONE-Paper.docx [file pone.0334417.s009.docx]

Network Pharmacology Approach Identifies Novel Anticancer Botanicals: Experimental Validation of Falcaria vulgaris (Sickleweed) as a therapeutic Candidate

Summary:

The authors identified potential anticancer botanicals using a network analysis from metabolites of thousands of medicinal plants. They shortlisted several candidates including *F. vulgaris*. They evaluated the potential anticancer effect of *F. vulgaris* extract on 2 breast cancer cell lines. The network analysis and metabolite information can be useful in discovering new plant-based cancer therapies. However, it is not clear how it can prevent cell growth and whether the effect observed is only in cancer cell lines and not in normal cells. Overall writing style and paragraph structure should be improved.

Major points:

- Overall concentrations of extract used are very high, more control experiments are needed to verify if the cell viability and anti-cancer activity is not just toxicity due to high concentration of extract. Please clarify whether the negative control used is the solvent used for the extract.
- Include which statistical analysis was performed for each figure in the respective figure legend.
- Figure 3: Not clear what control is, just cells with no compounds, a negative control of whatever solvent was used for the extract should be included. Also presenting the data in a dose response curve format would be better, showing concentration in the x axis. Also, it would be useful that once data is presented in a dose curve format the IC50 value is calculated.
- Figure 4: Again, it is not clear what control means, control should be the same amount of the extract solvent used.
- It would be nice to include in the introduction why extracts from plants are better than synthetic drugs used for cancer therapies, specifically targeted therapies. Are there any examples in the literature that show that plant extracts have less side effects than targeted therapies? Please elaborate on that.
- It is probably best to state that this is an exploration of *F. vulgaris* as a therapeutic candidate and not validation, as the exact mechanism of how is inhibits cell proliferation is not elucidated.

Minor points:

- Line 46. What is the evidence that metabolites don’t cause side effects on humans: Cite source and give example or remove sentence.
- Line 61. “Otherwise….” Simplify sentence for better readability.
- Line 62: “graph” features. Please be clear, what does graph features mean?
- Line 68: Why did they select Breast Cancer to test the anti-cancer properties, why not other cancers? Please elaborate on this on the intro. What kind of therapies are being used currently for Breast cancer.
- Figure 1: A, B and C: Please provide a legend for the color and size of each data point on the figure not only in the legend. D: Please explain better what “Replicate” means in the figure legend.
- Line 194: There is no figure 1e, please fix.
- Line 180: What is the relevance of that sentence in that paragraph.
- Line 232: Please explain better how the IC50 was calculated, it is not clear from the bar graph.
- Line 308: Change “methods” for approaches.
- Line 309: Fix grammar of sentence.
- It would be interesting to know from the metabolites found in the extract of *F. vulgaris*, which is the one that exhibits the major anti-cancer effect and if there is anything known in literature of how it prevents cell proliferation. Is it by inhibiting a specific oncogenic protein or is it affecting a cell growth pathway?
