# Supplementary material for: Network pharmacology approach identifies novel anticancer botanicals: Experimental exploration of Falcaria vulgaris (Sickleweed) as a therapeutic candidate
Source: PLoS One. 2026 Feb 27;21(2):e0334417. doi: 10.1371/journal.pone.0334417 (PMC12948079; doi:10.1371/journal.pone.0334417)
Supplement: Attachment — Submitted filename: Response to Reviewers.docx [file pone.0334417.s011.docx]

**Manuscript PONE-D-25-27242**

**Response to Reviewers**

Dear Prof. Dr. Awatif Abid Al-Judaibi,

Thank you for considering our manuscript “*Network Pharmacology Approach Identifies Novel Anticancer Botanicals: Experimental exploration of Falcaria vulgaris (Sickleweed) as a therapeutic Candidate*’’ for publication in the PLOS ONE. We sincerely appreciate the time and effort invested by you, and the reviewers in evaluating our work. We have carefully considered all feedback and are committed to addressing every concern in the revised manuscript. As requested, all modifications are highlighted in the manuscript using Track Changes. A point-by-point response to the reviewers’ comments is provided below (in blue text), detailing how each suggestion was addressed. Line and page numbers referenced correspond to the revised manuscript with inline revisions.

We hope the revised manuscript now meets the journal’s standards and look forward to your further guidance. Thank you again for your consideration.

*Fig.1 revised with color-size scaling, two supplementary Tables (S3 and S4) have been added.

** References number #79-82 and #84-86 have been added in the revised version as new references.

**Comments to the Authors:**

**A. Journal Requirements:**

When submitting your revision, we need you to address these additional requirements.

1. Please ensure that your manuscript meets PLOS ONE's style requirements, including those for file naming. The PLOS ONE style templates can be found at https://journals.plos.org/plosone/s/file?id=wjVg/PLOSOne_formatting_sample_main_body.pdf and https://journals.plos.org/plosone/s/file?id=ba62/PLOSOne_formatting_sample_title_authors_affiliations.pdf

**Author Response:** We confirm the manuscript now fully complies with PLOS ONE's style requirements, including: (1) proper file naming conventions (e.g., "S1_Table_RawData.xlsx", "Fig1.tif"); (2) 14 to 18-pt Time New Roman font (Heading 1-3) with double line spacing and continuous line numbering; (3) clearly labeled supporting information files (S1, S2, etc.) in approved formats; and (4) adherence to all relevant reporting guidelines. We have verified compliance using the PLOS ONE submission checklist and stand ready to address any additional formatting requests.

2. Your ethics statement should only appear in the Methods section of your manuscript. If your ethics statement is written in any section besides the Methods, please move it to the Methods section and delete it from any other section. Please ensure that your ethics statement is included in your manuscript, as the ethics statement entered into the online submission form will not be published alongside your manuscript.

**Author Response:** We appreciate the Editor’s careful attention to this detail. As requested, we have relocated the ethics statement exclusively to the Method’s section “Cell culture, MTT assay and Flow cytometry” in page 9, lines 189-191, and removed any duplicate mentions of the ethics statement from other sections of the manuscript.

3. Please amend your list of authors on the manuscript to ensure that each author is linked to an affiliation. Authors’ affiliations should reflect the institution where the work was done (if authors moved subsequently, you can also list the new affiliation stating “current affiliation: ….” as necessary).

**Author Response:** We have carefully revised the author affiliations to ensure every author is linked to at least one primary affiliation (the institution where their contribution to the work was conducted, e.g., Department of Biology, Faculty of Science, Hakim Sabzevari University, Sabzevar, Iran). We checked superscript numbers to associate each author with their correct affiliation(s) in the manuscript title page. For authors with multiple affiliations, we retained all relevant institutions on the title page per PLOS ONE’s guidelines, but marked the primary affiliation (where the research was performed) first, followed by secondary affiliations where applicable. These changes now fully comply with PLOS ONE’s requirements, and the affiliations are clearly linked to each author in the revised manuscript.

4. Please include captions for your Supporting Information files at the end of your manuscript, and update any in-text citations to match accordingly. Please see our Supporting Information guidelines for more information: http://journals.plos.org/plosone/s/supporting-information.

**Author Response:** We appreciate the guidance on formatting Supporting Information (SI) files. We have made the following revisions to ensure full compliance with PLOS ONE’s requirements:

A. Added detailed captions for all Supporting Information files (S1 File, S2 File, etc.) in a dedicated "Supporting Information" section at the end of the manuscript, just before the Declarations.

B. Updated all in-text citations to use PLOS ONE’s preferred format (e.g., "S1 Fig.", "S2 Table" instead of "Supplementary Figure 1").

C. Verified consistency with the journal’s Supporting Information guidelines regarding file naming and content organization.

These changes have been implemented throughout the manuscript, and we confirm that all Supporting Information files are properly cited and described.

5. Please review your reference list to ensure that it is complete and correct. If you have cited papers that have been retracted, please include the rationale for doing so in the manuscript text, or remove these references and replace them with relevant current references. Any changes to the reference list should be mentioned in the rebuttal letter that accompanies your revised manuscript. If you need to cite a retracted article, indicate the article’s retracted status in the References list and also include a citation and full reference for the retraction notice.

**Author Response:** We sincerely appreciate the Editor’s diligence and valuable feedback regarding reference accuracy. We have added the missing citation for reference #35 in the manuscript text (Page 10, line 206). To ensure reference integrity, we conducted comprehensive verification of all 86 references through PubMed, Retraction Watch Database, and journal websites (as of July 2024). No retracted papers were identified. Older references (e.g., #33, 1955 enzyme assay) were retained due to their established methodological role and no retractions were found.

We confirm the reference list is complete, accurate, and free of retracted works. Thank you for highlighting this important aspect of manuscript preparation.

**B. Reviewer #1:** This manuscript explores an integrative approach where network pharmacology and experimental validation are combined to search for novel anticancer botanicals, specifically focusing on *Falcaria vulgaris* (Sickleweed). The authors constructed a bipartite network of 3,250 plant species and 667 experimentally validated anticancer metabolites, resulting in the identification of 61 top-ranked plants. Among these, 85.25% had previously reported anticancer properties, and 14.75% were new candidates. For experimental validation, *F. vulgaris* was selected, showing significant cytotoxic and apoptotic effects on breast cancer cell lines (MCF-7 and 4T1). It also consisted of bioactive ingredients possessing known antioxidant, anticancer, and anti-inflammatory activities.

**Author Response:** We sincerely thank the reviewer for their thoughtful evaluation and constructive comments on our manuscript. Their insightful observations regarding the network pharmacology approach and experimental validation of *Falcaria vulgaris* will certainly help improve the quality and clarity of our work. We have carefully addressed all points raised, as the reviewer's feedback has been invaluable in refining this study.

But there are some weaknesses Points:

1. The paper requires detailed explanation of network analysis:

-Criteria for selection of metabolites and construct networks,

**Author Response:** We appreciate the reviewer's valuable suggestion to elaborate on our network methodology. In response, we have expanded Section "Anticancer Metabolite List" to provide greater transparency about our approach (Page 6, lines 116-126). Specifically, we now detail: (1) the systematic database searches (PubChem, KNApSAcK) using validated keywords ("anticancer", "anti-tumor", "cytotoxic"); (2) our three-tier inclusion criteria requiring peer-reviewed experimental evidence, confirmed plant origin, and documented mechanisms of action; and (3) the construction of an unweighted bipartite network in Cytoscape 3.9.1 linking the 667 validated metabolites to their 3,250 host plants. The complete metabolite list is provided in Supplementary Table 1. While our focus was on compiling experimentally verified relationships rather than computational validation, we acknowledge this as an interesting direction for future methodological development. These clarifications strengthen the reproducibility of our study while maintaining its biological focus.

-Algorithms and parameters used to rank plants, validation metrics for network predictions (e.g., sensitivity, specificity, cross-validation).

**Author Response:** We sincerely appreciate the reviewer's valuable suggestions for improving methodological transparency. We have substantially revised Section “Network Analysis” to provide: (1) full details of the eight topological parameters used for plant ranking (Degree, Betweenness, Closeness, Edge percolated component, Eccentricity, Radiality, Stress, and Bottleneck scores calculated via CytoHubba/Network Analyzer), (2) a three-tier validation approach combining prospective experimental testing of *F. vulgaris* (selected when no anticancer reports existed) with retrospective literature validation to concordance with known anticancer species, and (3) explicit discussion of methodological considerations including our unweighted network approach. These additions (Page 6, Lines 131-143; Supplementary Table S3) provide complete transparency about our ranking algorithm while demonstrating its predictive value through successful experimental confirmation of *F. vulgaris's* anticancer properties.

2. Why did you select *Falcaria vulgaris* for experimental validation over the other novel candidates identified in your analysis?

**Author Response:** We selected *Falcaria vulgaris* for experimental validation based on the following considerations:

1. Novelty with supporting evidence: while our network analysis identified nine new potential anticancer plants (including *Diospyros kaki*, *Angelica furcijuga*, and *Juniperus thurifera*), *F. vulgaris* stood out due to:

Page 5, line 105-107; Traditional Use: documented therapeutic properties in ethnomedicine (antioxidant, antimicrobial, anti-inflammatory) [#21, #22]

Page 5, line 107-110; phytochemical richness: high abundance of bioactive compounds with known anticancer potential (e.g., flavonoids, caryophyllene oxide, spathulenol) [#23–#26]

Page 5, line 110-112; GC-MS Validation: confirmed presence of key anticancer-associated components (carvacrol, α-pinene) [#26]

2. Strategic Prioritization:

Page 5, line 112-114; Research Gap: Despite its phytochemical profile*, F. vulgaris* had limited direct evidence for anticancer effects, making it an ideal candidate for experimental validation.

Regional Relevance: As a native Iranian species, it aligned with our goal to explore understudied medicinal plants with local availability for future translational work.

3. Comparative Advantages: stronger preliminary data (vs. other novel candidates) from traditional use and phytochemistry, as well as higher feasibility for laboratory validation (extraction yields, compound stability)

We acknowledge that other eight candidates (e.g., *Sedum takesimense*) also show promise and merit future investigation (page 23, line 413-425). This rationale clarifies our selection process.

3. Did you test the cytotoxicity of *F. vulgaris* extract on any normal (non-cancerous) cell lines to assess selectivity and potential toxicity?

**Author Response:** We appreciate this important question regarding selectivity and potential toxicity. In addition to our cancer cell line testing, we evaluated *F. vulgaris* extract cytotoxicity in normal 3T3 fibroblast cells (Swiss albino mouse embryo origin) as a preliminary assessment of therapeutic window. The extract showed no significant growth inhibition (p>0.05) in normal cells at concentrations up to 30 mg/mL (MTT assay, 24 h exposure), while demonstrating potent, dose-dependent cytotoxicity in cancer cell lines (more than 90% cell death in 30 mg/mL). This preliminary selectivity suggests cancer-specific activity, though we acknowledge that further testing in additional normal human cell lines would strengthen these findings.

4. The highest concentrations used in cytotoxicity assays (up to 10 mg/mL) are rather high; discussion about physiological extensibility and possible toxicity in vivo is warranted.

**Author Response:** We appreciate this insightful observation regarding our extract concentrations. While 10 mg/mL exceeds typical plasma levels, this aligns with in vivo safety data from Goorani et al. (2019) showing *F. vulgaris* tolerability at 200 mg/kg in rats, Basatinya et al. (2021) reporting efficacy of 500 mg/kg ethanolic extract of *F. vulgaris* for 14 days in rats, and Jafari et al. (2022) reporting comparable in vitro doses (22.5 mg/mL). The higher concentrations were necessary due to crude extract limitations (only 22.97% active palmitic acid; reduced bioavailability) and because lower doses (0.15-2.5 mg/mL) showed no cytotoxicity in MTT/flow cytometry assays. Preliminary mouse studies (1500 mg/kg, unpublished) further support safety. While future pharmacokinetic studies will refine therapeutic windows, our selected doses (≥5 mg/mL) successfully established proof-of-concept activity against MCF-7/4T1 cells, as detailed in Results (Page 18, lines 338-347).

5. Twenty-two compounds were obtained by GC-MS analysis, but there are some questions that the manuscript should meet:

-What specific compounds the anticancer effect bring-about. If the active compounds were fractionated or isolated.

**Author Response:** We thank the reviewer for prompting this important clarification. Our GC-MS analysis identified 22 compounds in *F. vulgaris* extract, with two dominant classes collectively representing ~80% of the composition and demonstrating validated anticancer mechanisms: (1) Fatty acids/steroids (49.56%, e.g., n-Hexadecanoic acid and Stigmasterol), and (2) Terpenes (30.2%, e.g., Phytol and Spathulenol). The two most abundant bioactive compounds, n-Hexadecanoic acid (22.97%) and Phytol (16.69%), which collectively representing ~40% of the extract are strongly associated with the observed bioactivity. n-Hexadecanoic acid demonstrated dose-dependent cytotoxicity in breast cancer cells via FAK/AKT pathway inhibition (Deepti et al. 2024). The n-hexadecanoic acid-enriched extract of *Halymenia durvillei* promotes apoptosis and autophagy of human triple-negative breast cancer cells (Sangpairoj et al. 2022). *Citrus unshiu* leaf extract containing phytol as a major compound induces autophagic cell death in human gastric adenocarcinoma AGS cells (Song et al 2015), and its synergistic effects with stigmasterol (another major component in *F. vulgaris*) enhance anticancer activity. While these two components are primary candidates for the observed effects, we acknowledge that minor constituents (e.g., spathulenol, stigmasterol) may contribute to the extract's overall efficacy through potential synergistic interactions.

While we have not yet fractionated individual compounds (a limitation noted in page 27 line 493-495), the extract’s efficacy aligns with reported bioactive concentrations (page 25-27, line 455-492).

-If possible, comparison with known concentrations of these compounds in other anti-cancer studies.

**Author Response:** Thank you for this suggestion. Our comparison of key F. vulgaris compounds with concentrations reported in other anticancer studies shows similar trends. For example:

A. n-Hexadecanoic acid (palmitic acid) constituted 22.97% of our extract, equivalent to ~2.3 mg/mL (9 mM) at 10 mg/mL crude extract

B. Phytol represented 16.69% of our extract, equaling ~1.7 mg/mL (5.7 mM) at 10 mg/mL crude extract

Wang et al. (2023) reviewed the molecular mechanisms of palmitic acid and its derivatives in tumor progression, demonstrating that palmitic acid affects various cancer cell lines through proliferation, apoptosis, metastasis, metabolic reprogramming, and autophagy across concentrations ranging from 5 μM to 50 mM. The dose closest to our study (10 mM) was shown by Beeharry et al. (2004) to increase oxidative stress through ROS generation, inhibiting RINm5F cell growth and promoting apoptosis.

Regarding phytol, a literature review (Islam et al., 2023) reports an effective treatment range of 3-400 μM. However, some studies required extended treatment durations (e.g., 40 μM for 7 days; Soltanian et al., 2020). Other investigations, such as Thakor et al. (2017), used higher concentrations (exceeding those in our study) and demonstrated phytol's cytotoxicity against human lung carcinoma (A549) cells, with an IC_50_ of 16.97 ± 2.31 mM, and observed the induction of bleb-like apoptotic bodies.

Overall, while the concentration range applied in our study is relatively high, we selected doses ≥5 mg/mL of crude extract for the MTT and flowcytometry assays because lower concentrations (0.15, 0.31, 0.62, 1.25, and 2.5 mg/mL crude extract) did not show significant inhibitory effects on MCF-7 and 4T1 cell lines compared to the control (0 mg/mL extract).

**C. Reviewer #2:**

The authors identified potential anticancer botanicals using a network analysis from metabolites of thousands of medicinal plants. They shortlisted several candidates including *F. vulgaris*. They evaluated the potential anticancer effect of *F. vulgaris* extract on 2 breast cancer cell lines. The network analysis and metabolite information can be useful in discovering new plant-based cancer therapies. However, it is not clear how it can prevent cell growth and whether the effect observed is only in cancer cell lines and not in normal cells. Overall writing style and paragraph structure should be improved.

**Author Response:** We sincerely appreciate the reviewer’s valuable feedback, which has helped strengthen our manuscript. In response to the all major and minor comments, specially we have: (1) clarified the mechanistic basis of F. vulgaris metabolites by detailing their predicted interactions with key cancer pathways (apoptosis regulators, cancer stem cell markers, Bcl-2, cell cycle proteins CDK4/6 and cell cycle regulation), supported by additional references; (2) included and highlighted our preliminary cytotoxicity examination on normal 3T3 cells; (3) improved figure legends for better clarity; (4) enhanced the literature review regarding plant-derived anticancer agents; and (5) improved writing style. We hope these revisions and cross-referenced responses adequately address all concerns and have improved both the scientific rigor and readability of our work.

Major points:

1- Overall concentrations of extract used are very high, more control experiments are needed to verify if the cell viability and anti-cancer activity is not just toxicity due to high concentration of extract. Please clarify whether the negative control used is the solvent used for the extract.

**Author Response:** We appreciate the opportunity to clarify these critical methodological points. The higher concentrations (≥5 mg/mL) were necessary because lower doses (0.15-2.5 mg/mL) showed no cytotoxicity in MTT/flow cytometry assays and due to crude extract limitations (only 22.97% active palmitic acid; reduced bioavailability). While future pharmacokinetic studies will refine therapeutic windows, our selected doses (≥5 mg/mL) successfully established proof-of-concept activity against MCF-7/4T1 cells, as detailed in Results (Page 18, lines 338-347). On the other hand, these concentrations align with in vivo safety data from Goorani et al. (2019) showing *F. vulgaris* tolerability at 200 mg/kg in rats, Basatinya et al. (2021) reporting efficacy of 500 mg/kg ethanolic extract of *F. vulgaris* for 14 days in rats, and Jafari et al. (2022) reporting comparable in vitro doses (22.5 mg/mL).

Regarding to the solvent used for the extract, we should clarify that the hydroalcoholic extract (70% ethanol) was completely dried and reconstituted in sterile ddH₂O for treatments (page 7, line 148-153), ensuring No residual ethanol, and identical solvent conditions between treatment and control groups (sterile water as negative control).

2- Include which statistical analysis was performed for each figure in the respective figure legend.

**Author Response:** Thank you for this suggestion. We have now explicitly stated the statistical methods in each figure legend as follows: Figure 2 (Antioxidant assay), “Data is represented as Mean ± SD, n = 3.”; Figure 3 (MTT assay), “Data analyzed by one-way ANOVA with Tukey test (n=4 replicates; **p<0.0001 vs control)”; and Figure 4 (Flow cytometry assay), “Numbers in each quadrant reflects the percentage of cells (population).”.

Analyses were performed using GraphPad Prism 8 with α=0.05. These updates appear in the revised manuscript figure legends.

3- Figure 3: Not clear what control is, just cells with no compounds, a negative control of whatever solvent was used for the extract should be included. Also presenting the data in a dose response curve format would be better, showing concentration in the x axis. Also, it would be useful that once data is presented in a dose curve format the IC50 value is calculated.

**Author Response:** We appreciate this opportunity to clarify our controls. In Figure 3 (MTT assay): the "Control" (0 mg/mL extract) represents cells treated with the identical solvent used for the extract (sterile ddH₂O), same volume as in treated groups, and identical incubation conditions. To clarify this, we changed "0 mg/ml extract (C, as control)" to "0 mg/ml extract (C, solvent control, same volume ddH₂O as in treated groups)" in figure legend. Also, mentioned details were added to the Methods (*italic* texts):

Page 10, line 202-205: … the cells were treated with different concentrations of extract of F. vulgaris (*0 mg/ml as solvent control contains same volume ddH2O with in treated groups,* 0.15, 0.31, 0.62, 1.25, 2.5, 5, 10, 20, 30, 40, 50, 60, 70, 80 and 90 mg/ml) for 24, 48, and 72 hours.

Regard to presenting the data in a dose response curve format and calculating IC50 value, we appreciate the suggestion to present cytotoxicity data as dose-response curves. While we agree this format effectively illustrates IC50 calculations, we have retained the column charts as the current column format (Figure 3) directly compares effects across multiple concentrations vs. control in cancer cell lines, and all calculated IC50 values are now explicitly stated in the revised text and S4 Table (MCF-7: 5,32 mg/mL; 4T1: 5.99 mg/mL in 24h incubation period) at page 18, line 345-347. As a compromise, we have extracted dose-response curves (with log-transformed x-axes) and results for three incubation period were presented as Supplementary Table S4.

4- Figure 4: Again, it is not clear what control means, control should be the same amount of the extract solvent used.

**Author Response:** We appreciate this opportunity to clarify our controls. In Figure 4 (flow cytometry apoptosis assay): the "Control" (0 mg/mL extract) represents cells treated with the identical solvent used for the extract (sterile ddH₂O), same volume as in treated groups, and identical incubation conditions. To clarify this, we changed "0 mg/ml extract (C, as control)" to "0 mg/ml extract (C, solvent control, same volume ddH₂O as in treated groups)" in figure legend. Also, mentioned details were added to the Methods (*italic* texts):

Page 10, line 213-215: …were treated with F. vulgaris extract *(0 mg/ml as solvent control contains same volume ddH2O with in treated groups, 10 mg/ml, and 30 mg/ml)*.

5- It would be nice to include in the introduction, why extracts from plants are better than synthetic drugs used for cancer therapies, specifically targeted therapies. Are there any examples in the literature that show that plant extracts have less side effects than targeted therapies? Please elaborate on that.

**Author Response:** We sincerely appreciate this suggestion to strengthen our rationale for plant-based therapies. In the revised manuscript, we have expanded the Introduction (lines 46-51 with references #7-13 of original submission) with specific comparative advantages and reduced side effects of plant extracts:

Page 3-4, line 54-73 in revised version: "*Targeted therapies (e.g., kinase inhibitors) often face limitations due to single-pathway inhibition, leading to drug resistance and narrow therapeutic windows. In contrast, plant-derived extracts offer multi-target synergies that can simultaneously modulate proliferation, apoptosis, and oxidative stress pathways, potentially overcoming these limitations [7, 8]. Growing clinical evidence highlights their potential to improve safety profiles when combined with conventional therapies – for example, herbal interventions have been shown to significantly reduce chemotherapy-associated nausea, fatigue, preventing weight loss, relieves the gastrointestinal toxicity, and hematological toxicity in randomized trials [9, 10]. Controlled trials demonstrate plant extracts' superior safety profiles versus targeted therapies as a cross section study on clinical database reveals that plant extracts have less side effect in comparison to synthetic drugs, like Mistletoe (Viscum album) extract vs. erlotinib, Turmeric (Curcuma longo) extract vs. Bevacizumab, and Ginseng vs. Tyrosine kinase inhibitors [9]. This enhanced tolerability stems from natural buffering by complementary phytochemicals that protect non-target tissues while maintaining anticancer efficacy [11, 12]. Additionally, plant-based therapies demonstrate superior cost-effectiveness, with production costs for vinca alkaloids being orders of magnitude lower than synthetic biologics [13]. These advantages position plant extracts as promising complements to existing targeted therapies.*"

All claims are directly supported by our original references (#7–13).

6- It is probably best to state that this is an exploration of F. vulgaris as a therapeutic candidate and not validation, as the exact mechanism of how is inhibits cell proliferation is not elucidated.

**Author Response:** We appreciate this important clarification. As suggest by you, we changed the “validation” to “exploration” in the title and abstract. Retained “validation” specifically referring to confirming of network predictions and methodological validation of assays.

Title: “Network Pharmacology Approach Identifies Novel Anticancer Botanicals: Experimental ~~Validation~~ Exploration of *Falcaria vulgaris* (Sickleweed) as a therapeutic Candidate”.

Abstract: Our findings not only ~~validate~~ explore *F. vulgaris* as a promising candidate but also cover the way for systematic exploration of understudied botanicals in oncology.

Minor points:

1- Line 46. What is the evidence that metabolites don’t cause side effects on humans: Cite source and give example or remove sentence.

**Author Response:** We appreciate your valid concern. To clarify, our original text did not claim that plant-derived metabolites are entirely free of side effects. Rather, we highlighted their growing use as *alternative* therapeutic options due to their potential for fewer or mechanistically distinct adverse effects compared to conventional drugs. We have now revised the text to explicitly acknowledge that plant metabolites can exhibit side effects, while emphasizing their value in circumventing limitations like drug resistance:

Revised *text* (page 3, line 52-53): Consequently, there has been a rapidly increasing interest in exploring alternative approaches, including the utilization of medicinal plants and their active compounds, *which may offer distinct safety or tolerability advantages in specific contexts* [7–10].

2- Line 61. “Otherwise….” Simplify sentence for better readability.

**Author Response:** Thank you for this suggestion. The sentence has been simplified for clarity while retaining its original meaning:

Revised *text* (page 4, line 88-89): “*This approach avoids unnecessary costs from testing irrelevant plant species.*”

3- Line 62: “graph” features. Please be clear, what does graph features mean?

**Author Response:** We appreciate your request for clarification. The term "graph features" refers to quantifiable properties derived from network analysis, such as node degree, centrality measures, or clustering coefficients. We have revised the sentence to explicitly define these features for better readability.

Revised *text* (page 4, line 89-92): "A ranked list generated by analyzing the biological networks of plant metabolites*—using topological features (e.g., node centrality, connectivity, or radiality)—*becomes invaluable in such a scenario [20]."

4- Line 68: Why did they select Breast Cancer to test the anti-cancer properties, why not other cancers? Please elaborate on this on the intro. What kind of therapies are being used currently for Breast cancer.

**Author Response:** We appreciate your insightful question regarding our choice of breast cancer as a model system. Two key reasons informed this selection:

1. High prevalence and therapeutic challenges: as the most commonly diagnosed cancer worldwide, breast cancer treatment faces limitations with drug resistance and toxicity in current therapies (e.g., endocrine therapies, HER2-targeted drugs, and chemotherapies).

2. Established experimental models: breast cancer cell lines are well-characterized in pharmacological studies, offering standardized systems for initial anti-cancer activity screening.

Overall, given the study's objective to validate the anti-cancer potential of prioritized plants, the selection of breast cancer cell lines represents a scientifically justified model system.

As you suggest to elaborate about current therapies of breast cancer, we add below sentences to introduction; page 5, lines 98-104:

“*Standard breast cancer therapies include endocrine agents for hormone receptor-positive tumors, HER2-targeted biologics, and cytotoxic chemotherapy for triple-negative breast cancer (TNBC**), and emerging targeted therapies (CDK4/6 inhibitors, PARP inhibitors, immunotherapy) [4]. However, treatment resistance and toxicity limitations persist, motivating the search for novel phytochemical alternatives with distinct mechanisms of action.*”

5- Figure 1: A, B and C: Please provide a legend for the color and size of each data point on the figure not only in the legend. D: Please explain better what “Replicate” means in the figure legend.

**Author Response:** We thank you for these valuable suggestions to enhance figure interpretation. The figure and legend have been revised to include: (1) embedded color/size scale bars in panels A-C (generated via Cytoscape's Legend Creator plugin) alongside the figure legend, and (2) clearer explanation of key terms (replicate). The updated legend now specifies that node size and color gradient (green [degree=1] to red [degree=244]) represent connectivity in the bipartite network, while panel D's "replicate" explicitly denotes how often each plant appeared in top-100 rankings across all network analyzing parameters.

6- Line 194: There is no figure 1e, please fix.

**Author Response:** Thank you for pointing this out. The text was changed to “*figure 1d*”, page 14, line 268.

7- Line 180: What is the relevance of that sentence in that paragraph.

**Author Response:** Thank you for this constructive comment. The sentence has been revised to better integrate with the paragraph's focus on metabolite distribution patterns while providing clearer scientific justification; page 12, line 247-251:

"*The quantitative distribution of these anticancer metabolites across plant species is particularly noteworthy, as phytochemical synergy research suggests that plants containing multiple bioactive compounds may offer enhanced therapeutic potential through multi-target effects* [14].”

8- Line 232: Please explain better how the IC50 was calculated, it is not clear from the bar graph.

**Author Response:** We appreciate your request for clarification regarding IC50 determination. The IC50 was calculated from dose-response curves of radical scavenging activity (RSA). Serial dilutions of each extract were tested, with concentrations plotted on the x-axis and corresponding RSA percentages on the y-axis. A linear regression model (y = mx + b) was fitted to the data, from which the IC50 was mathematically derived as the x-value at y = 50% RSA. Lower IC50 values indicate greater antioxidant potency, representing the extract concentration required to neutralize 50% of free radicals.

To better clarify IC50 in the text, below sentence was added; page 17, line 317-319:

“*The IC50, representing the extract concentration required to neutralize 50% of free radicals, was determined from dose-response curves of RSA using linear regression analysis of the concentration-RSA relationship.*”

9- Line 308: Change “methods” for approaches.

**Author Response:** We thank you for this helpful suggestion. The text has been revised as requested, with "methods" changed to "approaches", page 23, line 409.

10- Line 309: Fix grammar of sentence.

**Author Response:** Thank you for pointing this out. The sentence grammar was fixed and edited as below; page 23, line 410-412:

“*In addition to experimentally confirmed anticancer plants, our results remarkably included nine novel, previously undocumented anticancer plants (Table 3), based on literature reviews conducted during this study*.”

11- It would be interesting to know from the metabolites found in the extract of F. vulgaris, which is the one that exhibits the major anti-cancer effect and if there is anything known in literature of how it prevents cell proliferation. Is it by inhibiting a specific oncogenic protein or is it affecting a cell growth pathway?

**Author Response:** We thank you for prompting this important clarification. Our GC-MS analysis identified 22 compounds in *F. vulgaris* extract, with two dominant classes collectively representing ~80% of the composition and demonstrating validated anticancer mechanisms: (1) Fatty acids/steroids (49.56%, e.g., n-Hexadecanoic acid and Stigmasterol), and (2) Terpenes (30.2%, e.g., Phytol and Spathulenol). The two most abundant bioactive compounds, n-Hexadecanoic acid (22.97%) and Phytol (16.69%), which collectively representing ~40% of the extract are strongly associated with the observed bioactivity. Wang et al. (2023) reviewed the molecular mechanisms of n-Hexadecanoic acid (palmitic acid) and its derivatives in tumor progression, demonstrating that palmitic acid affects various cancer cell lines through proliferation, apoptosis, metastasis, metabolic reprogramming, and autophagy. Beeharry et al. (2004) also showed palmitic acid increase oxidative stress through ROS generation, inhibiting RINm5F cell growth and promoting apoptosis. As a major compound of the extract, n-Hexadecanoic acid demonstrated dose-dependent cytotoxicity in breast cancer cells via FAK/AKT pathway inhibition (Deepti et al. 2024). The n-hexadecanoic acid-enriched extract of *Halymenia durvillei* promotes apoptosis and autophagy of human triple-negative breast cancer cells (Sangpairoj et al. 2022).

Regarding phytol, Soltanian et al., (2020) show that phytol can down-regulate the expression of cancer stem cell markers (e.g., OCT4, NANOG, SOX2, ALDH1, ABCB1, CD44 and CD133) and reduce the proportion of side population cells in NCCIT human embryonic carcinoma cells. Other investigations, such as Thakor et al. (2017), demonstrated phytol's cytotoxicity against human lung carcinoma (A549) cells and observed the induction of bleb-like apoptotic bodies. *Citrus unshiu* leaf extract containing phytol as a major compound induces autophagic cell death in human gastric adenocarcinoma AGS cells (Song et al 2015), and its synergistic effects with stigmasterol (another major component in F. vulgaris) enhance anticancer activity.

While these two components are primary candidates for the observed effects, we acknowledge that minor constituents (e.g., spathulenol, stigmasterol) may contribute to the extract's overall efficacy through potential synergistic interactions. On the other hand, despite the fact that our phytochemical profiling identified these compounds, the specific contribution of each metabolite to *F. vulgaris*’s observed anticancer effects requires further validation through compound isolation and individual testing.

We have added this mechanistic context to the Discussion (page 25, line 458-478) and highlighted the need for future target identification studies (page 27, line 493-495).

**D. Reviewer #3:** The authors provide a clear and concise description of the problem they intended to solve and the objective of their study in the introduction. The methods employed are also clearly stated and align with the results obtained. The manuscript is also presented in clear and concise English language.

**Author Response:** We sincerely appreciate your thoughtful evaluation of our manuscript. Thank you for recognizing the clarity of our problem statement and study objectives in the introduction, the alignment between our methodological approach and the results obtained, and the conciseness and readability of the English language presentation. We have maintained these strengths throughout the revised manuscript while incorporating all suggested improvements from the review process.

**References Cited in Response to Reviewer Comments:**

Wang X, Zhang C, and Bao N. Molecular mechanism of palmitic acid and its derivatives in tumor progression. Frontiers in Oncology. 2023; 13:1224125.

Beeharry N, Chambers JA, Green IC. Fatty acid protection from palmitic acid-induced apoptosis is lost following PI3-kinase inhibition. Apoptosis. 2004; 9(5):599-607.

Deepthi K, Rahman H, Renjith PK. et al. Structural and morphological characterization, anticancer and antimicrobial study of 20EA column fraction in Sesbania grandiflora. Vegetos. 2024; https://doi.org/10.1007/s42535-024-01117-6.

Sangpairoj K, Settacomkul R, Siangcham T, Meemon K, Niamnont N, Sornkaew N. et al. Hexadecanoic acid-enriched extract of Halymenia durvillei induces apoptotic and autophagic death of human triple-negative breast cancer cells by upregulating ER stress. Asian Pacific Journal of Tropical Biomedicine. 2022; 12(3): 132-140.

Soltanian S, Sheikhbahaei M, Ziasistani M. Phytol Down-Regulates Expression of Some Cancer Stem Cell Markers and Decreases Side Population Proportion in Human Embryonic Carcinoma NCCIT Cells. Nutrition and Cancer. 2020; 73(8), 1520–1533.

Thakor P, Subramanian RB, Thakkar SS, Ray A, Thakkar VR. Phytol induces ROS mediated apoptosis by induction of caspase 9 and 3 through activation of TRAIL, FAS and TNF receptors and inhibits tumor progression factor glucose- 6- phosphate dehydrogenase in lung carcinoma cell line (A549). Biomedicine and Pharmacotherapie. 2017; 92: 491-500.

Song YW, Shrestha S, Gyawali R, et al. Citrus unshiu leaf extract containing phytol as a major compound induces autophagic cell death in human gastric adenocarcinoma AGS cells. Applied Biological Chemistry. 2015; 58, 257–265.
