# Supplementary material for: Network pharmacology approach identifies novel anticancer botanicals: Experimental exploration of Falcaria vulgaris (Sickleweed) as a therapeutic candidate
Source: PLoS One. 2026 Feb 27;21(2):e0334417. doi: 10.1371/journal.pone.0334417 (PMC12948079; doi:10.1371/journal.pone.0334417)
Supplement: Attachment — Submitted filename: Response_to_Reviewers_auresp_2.docx [file pone.0334417.s012.docx]

**Manuscript PONE-D-25-27242 R1**

**Response to Reviewers**

**Dear Prof. Dr. Awatif Abid Al-Judaibi,**

Thank you for the opportunity to revise our manuscript “*Network Pharmacology Approach Identifies Novel Anticancer Botanicals: Experimental exploration of Falcaria vulgaris (Sickleweed) as a therapeutic Candidate*’’ for publication in PLOS ONE. We are grateful to you and the reviewers for the insightful comments and constructive feedback, which have significantly strengthened our manuscript. We have carefully addressed all points raised in the latest round of review. Specifically, we have incorporated the following key revisions as requested:

1. Reviewer-Recommended Citations: We confirm that we have reviewed all reviewer suggestions and have only added new citations where they were directly relevant to support new claims or clarifications made during revision, in accordance with journal policy.

2. Reference Integrity: We have performed a new, comprehensive verification of our reference list against PubMed and can confirm that all citations are correct, complete, and none have been retracted.

3. Normal Cell Testing & Dose Rationale: Detailed descriptions of the methodology for the 3T3 normal cell line assay and the rationale for the chosen high-dose range have been added to the Materials and Methods section. The corresponding results are now presented in the Results section and visualized in S4 Fig.

4. *F. vulgaris* Selection Rationale: We have now clearly integrated the multi-faceted rationale for selecting *F. vulgaris* over other candidate plants into the Result, detailing its traditional use, phytochemical richness, and the strategic research gap it addresses.

All changes made to the manuscript have been meticulously highlighted using the Track Changes feature for your convenience. Our detailed point-by-point responses to all comments are included in the section below.

We believe that these revisions have fully addressed the editorial and reviewers' concerns and have markedly improved the clarity, rigor, and overall quality of our work. We are confident that the manuscript now meets the high standards of PLOS ONE.

Thank you again for your time and consideration. We look forward to your final decision.

Sincerely,

Eisa Kohan-Baghkheirati

*S4 Fig. has been added.

** Reference number #1 has been edited in the revised version based on the source paper.

**A. Journal Requirements:**

If the reviewer comments include a recommendation to cite specific previously published works, please review and evaluate these publications to determine whether they are relevant and should be cited. There is no requirement to cite these works unless the editor has indicated otherwise.

**Author Response:** We thank the Editor for this important reminder. We have carefully reviewed all reviewer comments from the previous round and confirm that none of the reviewers recommended the citation of specific previously published works. All new references added in our R1 revised manuscript were selected independently by us to support new text, clarify methods, or provide context for claims added during the revision process. Each citation was included solely based on its academic relevance to the enhancements made in the manuscript. We confirm that we have adhered to the journal's policy on this matter.

Please review your reference list to ensure that it is complete and correct. If you have cited papers that have been retracted, please include the rationale for doing so in the manuscript text, or remove these references and replace them with relevant current references. Any changes to the reference list should be mentioned in the rebuttal letter that accompanies your revised manuscript. If you need to cite a retracted article, indicate the article’s retracted status in the References list and also include a citation and full reference for the retraction notice.

**Author Response:** We thank the Editor for this critical reminder regarding reference integrity. In direct response to this comment, we conducted a meticulous, line-by-line verification of our entire reference list to ensure its completeness, correctness, and freedom from retracted publications. Each of the 93 references was individually checked against the PubMed database on September 8, 2025. Reference #1 was edited based on the source paper. We confirmed that none of the cited papers have been retracted. All references are valid and in good standing. We can therefore confirm that our reference list is complete, correct, and free of any retracted publications. No changes to the reference list were required as a result of this audit.

**B. Additional Editor Comments:**

Dear Authors,

Thank you for your submission to PLOS ONE. Based on the reviewers’ comments and your responses, please revise the manuscript to address the following:

1. Kindly, include details of the experiments performed to test the toxicity of *F. vulgaris* extract on non-cancerous cell lines, and provide the rationale for the chosen concentrations.

**Author Response:** We thank the Editor and reviewers (previous round) for this essential feedback. We have now integrated the requested details on the cytotoxicity assessment in normal cells and the rationale for dose selection directly into the Materials and Methods, and Results sections of the manuscript. The changes are as follows:

1. Materials and Methods (Page 10, lines 213-224): We have added details of specific normal cell line used (3T3 fibroblasts, Swiss albino mouse embryo origin), the culture conditions and assay used (MTT assay, 24h exposure), and the concentration range tested (0-90 mg/mL), aligning with the doses used on cancer cell lines. The rationale for using high concentrations, citing the crude nature of the extract, the low percentage of active compounds, and the precedent set by prior *in vivo* and *in vitro* studies on *F. vulgaris* (Ref. #22 and #23).

2. Results (Page 19, lines 375): We have added a new S4 Figure in the Results section presenting the findings from the normal cell assay, confirming the lack of significant cytotoxicity at concentrations up to 30 mg/ml, effective against cancer cells. This highlights the preliminary selective toxicity of the extract.

2. Clearly explain why *F. vulgaris* was selected over the other plants identified in the study. Please incorporate these points in the revised manuscript and resubmit for further consideration.

**Author Response:** We thank the Editor for this crucial feedback. We have now integrated a clear and detailed explanation for the selection of *F. vulgaris* for experimental validation directly into the Result section of the manuscript. This new paragraph outlines the multi-faceted rationale, balancing novelty with practical research considerations.

The new text (found on page 16, lines 316-333) explicitly states that *F. vulgaris* was selected based on:

1. Established Traditional Use & Bioactivity: Its documented ethnomedicinal properties (e.g., antioxidant, anti-inflammatory) provided a foundational safety and bioactivity profile.

2. Phytochemical Richness: GC-MS analysis confirmed a high abundance of metabolites with known anticancer associations (e.g., flavonoids, carvacrol, α-pinene).

3. Strategic Research Gap: It represented a high-potential but under-investigated candidate, allowing our study to generate novel, significant findings.

4. Feasibility and Translational Potential: As a regionally available species, it aligned with our long-term goal of exploring translatable therapeutic candidates.

5. We acknowledge the promise of the other highly-ranked plants from our analysis and have highlighted them as compelling candidates for future research.

**C. Reviewers Comments: -**

**Author Response:** All reviewer comments have been fully addressed in previous round. There are no new comments requiring a response at this stage.

We wish to express our sincere gratitude to Reviewers #1 and #2 for their time and effort in re-evaluating our manuscript. We are delighted that they found our revisions to be thorough and that the manuscript now meets the journal's standards for publication.

We also extend our thanks to the Editor for overseeing the review process. The constructive feedback provided throughout has been invaluable and has significantly strengthened the quality and clarity of our work.
